# An Experimental Inquiry into the Nature of Gravelly and Calculous Concretions in the Human Subject; and the Effects of Alkaline and Acid Substances on Them, in and out of the Body

**Published:** 1806-12

**Authors:** Thomas Egan


### *An experimental Inquiry into the Nature of Gravelly and Calculous Concretions in the Human Subject; and the Effects of Alkaline and Acid Substances on them, in and out of the Body. By* THOMAS EGAN, *M. D. M.R.I.A*

[ Concluded from our laſt, pp. 305—312. ]

EXP. 5.—To three ounces of the same kind of urine as in EXP. 4, was added one drachm of lime water: the result the same as in the former. Some precipitation in the standard, as in the preceding experiment.

When we consider the small proportion of lime kept in solution in water, and that the lime water used in my experiments was far from being recent, we must be astonished at the minute quantity that proves sufficient to keep the uric acid in solution: but this wonder ceases when we recollect that the proportion of it in the above quantities of urine is extremely small, and that it is scarcely acid; as we may learn from the controversy that took place between two such able chemists as Pearson and Fourcroy on this subject. Finding, then, our common lime water exerting such powers in preventing the separation or crystallization of this substance, it occurred to me, that much more might be expected from barytic lime water, as containing a larger proportion of saline matter in solution; and that, though, from its poisonous effects in the carbonated state, the internal exhibition would be hazardous, yet it might prove an useful remedy when injected into the blader. But how uncertain are our apparently best founded theories when not deduced from experiment!

EXP. 6.—To three ounces of urine was added one drachm of barytic lime water, which immediately seemed to decompose the whole, render it turbid, and give it the appearance of the *urina jumentosa*; for reasons easily and satisfactorily explained by Fourcroy, to whom I must refer. After two days I was surprised to find some small crystalline matter adhering to the sides of the glass; and, upon examining the copious precipitate from this re-agent, I found it blended with as much crystallized uric acid as appeared in the standard. A repetition of this experiment, both since and at that time, afforded the same results. Now the strength of barytic lime water is, to that of the common, nearly as 13 to 1; the former keeping in solution, at the temperature of 60 degrees, 13 grains to the ounce. Has the barytic, with all its superior energy, less affinity for the uric acid than the calcareous earth? or does a superiour affinity, to some other ingredients of the urinary compound, supersede its union to this?

I regret that the small quantity of stronthian lime water, which I possessed, did not permit me to extend my inquiries with it.

Finding, then, that our alkaline earth of lime, in the weakest possible state of solution, and in the smallest proportion, effectually prevents the crystallization, or keeps in solution the lithic acid of urine: if we only suppose that it reaches the kidneys and bladder in the smallest quantity, it must produce similar effects there, obviate the further formation of gravelly matter, or further accumulation of pre-existing calculous concretions of this kind.

Let us now proceed to inquire into the effects of the pure and carbonated alkalies themselves. The action of the former being well known and acknowledged, I shall content myself with one experiment, and pass on to the latter.

Exp. 7.—To four ounces of the urine of a child often depositing gravel, on cooling, were added ten drops of the *aqua kali puri* of the shops. After seven days no sign of separation: some observable in the standard after some hours.

Exp. 8.—To four ounces of urine were added three grains only of crystallized carbonate of potash, the purity of which was ascertained by Dr. Percival and myself, and containing, according to Mr. Kirwan, 1-23 grains of alkali. After several days no appearance of crystallized matter: some in the standard after forty-eight hours.

Exp. 9.—To the same quantity of urine were added two grains only of the same, with the like result.

From the pure and carbonated, let us now proceed to the super-carbonated and sub-carbonated states.

Exp. 10.—To three ounces of urine, in a well closed phial, was added half an ounce of the *aqua mephitica alkalina,* prepared according to Dr. Faulkner's proportions. No crystallized appearance after seven days. This result we might well expect, from the relative large proportion of its alkaline salt; having already seen equally good effects, from half an ounce only of Kinsley's soda water, containing a mere fraction of alkaline matter.

Exp. 11.—To three ounces of urine were added two grains of the common salt of tartar of the shops, containing, according to Mr. Kirwan, 0-82 of alkali. The same results as in the former experiments, even after six days.

Exp. 12.—Having no pure mineral alkali, three grains of the common crystallized soda of the shops, containing, according according to Mr. Kirwan, 0-64 of alkali, were added to four ounces of urine. The result as in the former; which was equally produced by two grains only; and perhaps would have been equally so by one.

Wishing to be more fully convinced that the very large proportion of disengaged carbonic acid gas in our soda waters did not counteract the usual alkaline effects:

Exp. 13.—To four ounces of the urine of the same child, which was generally surcharged with gravelly matter, I added half an ounce of Kinsley's soda water in its full state of effervescence: the phial well corked, and removed into a cold cellar, temperature 42 degrees. After four days, nay, a week, nothing but the usual calcareous sediment, without an atom of crystallized or otherwise precipitated uric acid.

From the above experiments, then, we learn, that pure lime in the state of lime water, the pure alkalies, the sub carbonated, carbonated, and super-carbonated, all prevent the separation of the uric acid, by uniting probably with and retaining it in solution. That they should still exert their power in the super-carbonated soda water, is rather singular; and we must suppose that, in the temperature of the human body, this superabundant gas (which, for the greater part, is only retained by compression) would be disengaged, and leave the alkali to exert its usual properties; and so, I would presume, it happens.

A half pint of soda water was poured into a large glass, and exposed to the influence of the atmosphere in a temperature of from 60 to 75 degrees. After two days it continued to turn litmus red, and only ceased to do so at the end of three. But in Experiment 10, we find it in its full gaseous state, still possessing its alkaline influence on the uric acid; which I would be disposed to attribute to its very weak union to the carbonic acid in the fully carbonated and super-carbonated states, as well as to the very weak degree of acidity of the uric acid itself, rendering the most minute portion of all alkaline matter sufficient to its saturation. However this may be, it is obvious that the extraordinary quantity of gas with which these waters are surcharged, is undoubtedly superfluous, and may probably prove dangerous. In gouty habits (so subject to these complaints) there is always danger of their inducing spasmodic affections of the stomach. This has frequently occurred; and if, to prevent it, we are obliged to add spirituous tinctures, and brandy, why not as well omit this super-saturation ration at once, and content ourselves with that pleasing degree of it which exceeds but little that of saturation?

Nor have the predisposed to apoplexy less to apprehend. And in these cases we find our own physicians, as well as those of the sister kingdom, preferring carbonated potash, or desiccated soda. But, recollecting that I am acting, on this occasion, the part only of the experimenter, I shall now proceed to consider what the action of these saline substances may be on the uric acid, in its concrete or calculous state, as well as on a few others of these concretions, which, though of a different nature, are of frequent occurrence, and easy solubility. The nature of those employed in the following experiments was always previously ascertained and specified; they were also carefully weighed and dried, both before and after immersion in their several menstrua.

Dr. Percival, of Manchester, as well as others, having experienced the solvent power of the plain mephitic, or carbonated water, on urinary calculi, it was thought proper to repeat his experiments.

Exp. 1.—A fragment of a calculus, weighing twenty-three grains, and of the uric acid kind, was suspended by a thread, for forty-eight hours, in Nooth's apparatus, already nearly filled with highly impregnated aërated water, and still exposed to a stream of carbonic acid gas: temperature 58 degrees. When taken out and dried, weighed, as before, twenty-three grains.

Exp. 2.—A fragment of a calculus, of the same kind, weighing forty grains and three quarters, was suspended, as before, in Nooth's apparatus, for forty-eight hours: temperature varying from forty to fifty-five degrees. On being taken out, and dried, was found to have sustained no loss.

Exp. 3.—An entire calculus, of a rough and sandy appearance, chiefly of the uric acid kind, but with some extremely minute intermixed particles of the ammoniacal magnesian phosphate, weighing fifty-two grains, was suspended, as before, for forty-eight hours, in Nooth's apparatus. After being taken out, and dried, was found to weigh fifty-one grains and a quarter; so that there was here a loss of three-quarters of a grain: undoubtedly of the ammoniacal magnesian phosphate.

Exp. 4.—Wishing to see whether even increased temperature would add to the solvent power of carbonic acid, a fragment of calculus, of the uric acid kind, weighing twenty-two grains, was immersed, as before, for forty-eight hours, in three ounces and a half of highly impregnated carbonated carbonated water, in a well closed phial, and laid on a sand heat which did not exceed the temperature of 100 degrees. After being taken out and dried, the weight was found as before, twenty-two grains.

From these experiments, then, we may conclude, that calculi of the uric acid kind are insoluble in carbonated water; and that Dr. Percival, whose character as a philosopher, as well as a physician, deservedly stands so high, must have operated upon concretions of a different kind; more especially as in his experiments there was a loss of several grains in only a few ounces of mephitic water, whilst none appeared in ours, though in several pounds of that fluid. He must have then operated upon some of a different and highly soluble kind.

Exp. 5.—One-half of a calculus, of the ammoniacal magnesian kind, weighing 100-5 grains, was suspended, as usual, for forty-eight hours in Nooth's apparatus: temperature 50 degrees. Upon being taken out, and dried, was found to weigh 92-63 grains so that its loss amounted to 7-87, or rather more than seven grains three-quarters. And here we have an explanation of the result of Dr. Percival's experiments, by snpposing that the calculi he employed must have been of this species; the extreme solubility of which, in so weak and innocent a menstruum, should excite the earnest hope of our young gentlemen, in the surgical department, of effecting its solution by injections of carbonated water into the bladder. To such safe trials they must be encouraged by the pleasing consideration that this kind forms a large proportion of the human urinary calculi. On these occasions the water should not be too highly impregnated, lest the sudden expansion of the gas under the human temperature might excite the bladder to reject it too quickly. This inconvenience, however, must be in part obviated by the necessity of previously warming the injection to about the temperature of 80 degrees; a precaution never to be omitted. But to return to the alkaline earths and salts:—

Exp. 6.—One-half of a uric acid calculus was suspended, for forty-eight hours, in four ounces of lime water: temperature as before. After being dried, was found to have lost seven grains three-quarters; and the surface to be covered with a granular efflorescence, which, in drying, detaches itself. The calculus was so much softened as to leave little doubt of its entire destruction by a few more immersions. It was again suspended for a month, in the same quantity of lime water, in the temperature of the at-

 mosphere mosphere only, without any renewal of the menstruum; when it was found to have lost twenty-four grains. Now, if the lime water had been frequently renewed, and its energy assisted by the standard heat of the human body, no doubt but it would have been entirely broken down in a much shorter time. We find, then, lime water not only preventing the separation of uric acid from urine, but acting powerfully upon it in its most compact form. How well founded, then, were the experiments of Whyte, as well as the opinion of Dr. Smyth; and how little deserving the latter the obloquy of his contemporaries for his predilection to it! Now this result, corresponding also with Scheele's, points it out to us as one of our most safe and active agents, when injected into the bladder with the necessary precautions. And we must feel surprised that no attempts of that kind have been made since the time of Whyte.

Exp. 7.—A fragment of calculus, of the uric acid kind, weighing seventy-nine grains and a quarter, was suspended, for forty-eight hours, in a mixture consisting of four ounces of distilled water and twenty-five drops of the weak *aqua kali puri* of the shops, which merely gave it an alkaline taste. It was then placed on a sand heat; temperature varying occasionally from 60 to 100 degrees. After being taken out, and dried, it weighed seventy-four grains and three-quarters; so that the loss amounted to four grains and a half. This weak lixivium, it appears, then, operated upon it, acquired a yellow colour, a sweet taste, and precipitated, with a few drops of muriatic acid, a white sediment, easily recognizable by the small, silky, needle-shaped, crystaline appearance peculiar to the uric acid.

Exp. 8.—This same fragment of calculus, after being well washed, and dried, was again immersed, for forty-eight hours, in a lixivium consisting of only twenty drops of *aqua kali puri* to four ounces of distilled water, and under the same circumstances. Upon being taken out, and dried, it was found to have lost four grains and a quarter. The solution was of a yellowish green colour, lost all alkaline taste, and precipitated, as before, with either the acetous or muriatic acid.

Exp. 9.—A fragment of calculus, of the same kind, weighing eighty-one grains, was suspended, as before, for forty-eight hours, in a mixture only of fifteen drops of the same alkali to four ounces of distilled water, which scarcely imparted an alkaline taste. After being taken out, and dried, it was found to have lost one grain and three-quarters; the specimen consisting chiefly of the external laminæ, much more slowly acted upon.

Exp.

Exp. 10.—This same fragment, washed and dried, was again immersed, for forty-eight hours, in a similar lixivium, and under the like circumstances. The loss now amounted to nearly four grains; and from this we learn how considerably the energy of the menstruum is increased by each succeeding immersion; so much so, indeed, that a few repetitions enable it to disunite the laminæ, and cause them to crumble into a pulverulent state, easily voidable with the urine. To the happy result, therefore, of this experiment let me earnestly solicit the attention of our young practitioners.

Exp. 11.—As children are such frequent sufferers, Mr. Richards suggested the propriety of ascertaining whether the alkaline influence might be weakened by the addition of sugar. One half of a calculus, of the uric acid kind, weighing 185½ grains, extracted by my friend Mr. Richard Dease, and (though under the most unpromising circumstances) with a dexterity and success not to be exceeded by his late father, was suspended in a lixivium consisting of eight ounces of distilled water and twenty drops of weak *aqua kali puri* (partly aërated), and scarce imparting an alkaline taste. To this were added thirty-six grains of sugar, which were found adequate to sweeten it sufficiently. After remaining forty-eight hours in a temperature varying from 55 to near 100 degrees, or a medium one of 74 degrees, being dried and weighed, it was found to lose ten grains three quarters. The addition, then, of saccharine matter cannot diminish, but may add to the alkaline energy.

Exp. 12.—Ten grains of very pure crystallized carbonate of potash were dissolved in four ounces of distilled water. In this filtered lixivium was suspended a fragment of calculus, of the uric acid kind, weighing seventy-two grains and a quarter, for forty eight hours, on a sand-heat, varying from 50 to 100 degrees (for the fire was not kept up during the night). Being taken out, dried, and weighed, it was found to have lost seven grains and a quarter. The solution had a yellowish green colour, different from the light yellow tinge of the pure alkaline ones. It also lost its taste, but without becoming sweet. A quantity of flocculent animal matter was separated, and the dissolved uric acid was, for the greater part, again precipitated, upon the mixture cooling, to the temperature of the atmosphere.

Exp. 13.—The crystallized carbonate of potash, being generally prescribed in the proportion of one drachm to four ounces of water; in a similar mixture was suspended an entire calculus, of a very compact, rough, and gritty appearance, weighing forty grains and a quarter. After remaining forty-eight hours in the above temperature, it was taken out, dried, and weighed, and found to have lost three grains three-quarters. The solution here more highly coloured than in the former: some spontaneous precipitation; and an immediate one, on the addition of a few drops of weak marine acid. We then find the vegetable alkali in the fullest state of saturation, with carbonic acid, that we can procure it, in the solid form, acting powerfully on these concretions, when assisted by degrees of temperature even much inferior to that of the human body.

Now, as to the mineral alkali, nature presents us with similar, nay, more extraordinary results, in the mild mineral alkaline impregnation of the waters of Carlsbad, in Bohemia. Here are several springs, varying in temperature from 114 degrees to that of the Brudel at 165 degrees. According to Elliot, they contain, in the gallon, of aërated lime 36 grains; muriate of soda 48; aërated soda 102; vitriolated soda 6 drachms; some minute proportion of iron, and a considerable carbonic acid impregnation. But Klaproth rates the proportion of mineral alkali still higher.

Of the lithontriptic effects of these waters, Springfield gives us a very surprising account indeed: founded, however, upon numerous experiments, instituted upon the spot, by the immersion of many calculi in the sources themselves; where they were either entirely dissolved, or acted upon with an energy that must appear incredible, if we did not consider the nature of the menstruum, its high temperature, and constant renewal by the flowing of the stream. Nay, the urine of patients who used these waters for a few days was found to possess powerful lithontriptic effects, as appeared by the immersion of many calculi in it. For an account of these highly interesting experiments, too numerous for insertion here, I must beg leave to refer to his Treatise. *De Prerogativa Thermarum Carolinarum, in dissolvendo Calculo Vesicæ, præ Aqua Calcis vivæ.*

From these experiments, as well as the highly beneficial effects of these waters, taken internally, by the numerous calculous and gravelly patients who frequent Carlsbad, he establishes their superiority over the different alkaline and other remedies hitherto in use, not excepting Whyte's oyster-shell lime water. Now, the lime in these being carbonated, bonated, and only kept in solution by their highly aërated state, we can be at no loss, in those days, to attribute their superior agency to the alkaline impregnation, assisted by so high a temperature. Klaproth affirms, that a person who drinks these waters, in the usual quantity, for twenty-six days, takes of mild mineral alkali 3913 grains, or 8 ounces 1 drachm and 13 grains; which amounts to two drachms and a half per day, besides the other saline ingredients.

Doctors Rutty and Smyth, who gave us a valuable extract from this publication, in the Memoirs of the Medical and Philosophical Society of this city, (now in the library of the Royal Irish Academy, but which, we have sincerely to regret, were never published, and are now discontinued,) conclude their account by the following query: "May not some alkaline lixivium be contrived by art, that would possess similar effects with these waters?" And has not this partly taken place in the instance of our soda waters? But may we not make a nearer approximation by a solution of the above specified proportion of mineral alkali in the relative quantity of water, with the addition or omission of the carbonic acid, and the other saline ingredients, as may be thought proper, afterwards heating, however, each separate dose to 160 degrees?

We find, then, the alkaline carbonates, in the great laboratory of nature, as well as in our experiments, exerting considerable solvent powers upon these animal concretions contrary to what has been hitherto supposed.

Exp. 14.—Into a filtered solution of ten grains of salt of tartar, in four ounces of distilled water, were introduced two fragments of calculi, weighing seventy-four grains and a quarter. The mixture was set aside for forty-eight hours in a cool room; temperature varying from 47 degrees at night, to 56 degrees in the day. After twelve hours it began to be coloured, and continued to be more so, until the temperature fell to 51 degrees, when a precipitation took place, and continued during the night; so that it appeared to deposit, at the temperature of 47 degrees, what was taken up at degrees somewhat exceeding 51°. These fragments, on being taken out, dried, and weighed, were found to have lost three grains and three quarters; the laminæ disposed to crack, and the strata to separate and crumble. This weak lixivium, then, exerted much energy, even in a very low temperature.

Exp. 15.—A fragment of calculus, weighing seventeen grains three-quarters, was immersed in a lixivium of similar strength; but now exposed to a temperature varying from

81 degrees at night to about 95 degrees in the day. After forty-eight hours, it was found to lose five grains and a half: a prodigious quantity, when we consider the small surface presented by this fragment, weighing only seventeen grains three-quarters. The solution, upon cooling, became turbid as before, and precipitated a large proportion of the dissolved uric acid.

Exp. 16.—A fragment of calculus, weighing forty grains three-quarters, was immersed in four ounces of soda water for forty-eight hours, and exposed to a temperature varying from 55 to about 100 degrees. Its loss amounted to one grain. A repetition of this experiment afforded nearly the same result; and demonstrates, that though the soda, in this super-carbonated state, still exerts some energy on concretions of the uric acid kind, yet it is but feeble; and that these waters appear more capable of preventing their formation than effecting their solution, when they once acquire the aggregate state. The same fragment, in a similar quantity of soda water, in the temperature of from 50 to 55 degrees only, sustained no loss, after forty-eight hours. And here we have another proof of the necessity of seriously attending to the degree of temperature in all researches of this kind.

But it may be observed, as to the internal use of alkaline substances in particular, that their effects must be considerably weakened upon their immediate admixture with the urine; as the small quantity that can be conveyed there must, in the first place, neutralize the uncombined phosphoric acid in all urine, the benzoic in children's, and decompose the ammoniacal and magnesian phosphates in that of every period of life. It must be acknowledged its efficacy is partly counteracted by these circumstances, which should never be overlooked, and always taken into account in practical application. Referring to Fourcroy's instructive essay on this subject, Memoirs of the National Institute, and *Connoissances Chimiques,* let us here once more appeal to the test of experiment.

Exp. 17.—A fragment of calculus, weighing eighteen grains one-quarter, of the uric acid kind, was suspended, for forty-eight hours, in an alkaline lixivium, consisting of four ounces and a half of recent urine, and twenty drops of a very weak, and partly aërated, caustic lixivium; medium temperature about 74 degrees. On being taken out, and dried, it was found to have lost one grain three-quarters; a considerable quantity from so small a specimen. To the filtered solution were added a few drops of dilute dilute marine acid, which, after a few minutes, precipitated a reddish crystalline matter in a triple proportion of what generally occurs in the natural state of urine.

From the above experiments, therefore, it appears no longer doubtful; first, that pure lime, even in the small proportion contained in lime water, and the pure alkalies, in an extreme state of dilution, in temperatures even somewhat inferior to those of the human system, exert an active solvent power on calculi of the uric acid kind: secondly, that the alkaline carbonates, under similar circumstances, are possessed of similar powers, though in an inferior degree: and thirdly, that, by our having ascertained this point, we have removed a long established error, substituted a discovery highly interesting to animal chemistry, and likely to be productive of a more enlightened and successful practice in the treatment of these diseases.

In these expectations we shall appear to be the better founded, when it is considered, that, for want of entire specimens (preserved here like the oriental bezoars of old), we were obliged to operate upon fragments presenting small surfaces only to our solvents: that these last were never renewed during the course of the experiments, which would not have occurred in their application in the form of injections; as they should, in that case, be so often repeated, and act, of course, with renewed energy: that, either taken internally, or used in form of injection, the smallest proportion of alkaline matter, in a great state of dilution, assisted by the human temperature, answers our purpose; and that the temperature in our experiments was never permanent, and might be rated at the medium one of 74 degrees.

Having now fulfilled the second object of this essay, I would no longer presume to trespass on the indulgence of the Reader, if I were not actuated by the sanguine hope of turning the attention of my surgical friends to the humane consideration of obviating, as much as possible, the most dangerous of operations by the prudent application of a few safe solvents injected into the bladder. How far they may succeed with calculi of the uric acid kind, may be already conjectured from the preceding experiments; but with those of the next most frequent occurrence there is much less difficulty to encounter, and every reason to hope for a speedy and safe result. The ammoniaco-magnesian phosphate is partly soluble in water, highly so in the carbonic acid (as we have already seen); and, consequently, more so in the weakest possible acid impregnations tions that can be advised; nothing more being necessary than the addition of as many drops only of weak muriatic acid as will scarce impart an acid taste. But as precept should, in every instance, be as much as possible assisted by experiment, I shall, for the encouragement of the young practitioner, exhibit a few on this very soluble species the more willingly, as he has no assistance to expect from his professional books; these subjects being only treated of in Philosophical Transactions, Memoirs of the National Institute, and a few other foreign chemical publications, if we except Whyte's Treatise on Lime Water, to which we would willingly refer him.

Exp. 18.—An entire calculus, of a reddish, gritty appearance, externally, proved to consist of ammoniaco-magnesian phosphate, weighing forty-six grains one quarter, was suspended, for forty-eight hours, in a mixture consisting of four ounces of distilled water, and ten drops of weak marine acid. After being taken out, and dried, it was found to have lost six grains three-quarters. The mixture was whitish, lost its acid taste, and precipitated, on the addition of a few drops of fixed alkali, the ammoniaco-magnesian phosphate, under that beautiful crystalline form so accurately described by Dr. Wollaston.

We may readily conceive how much more the loss would have amounted to in this case, in the short space of forty-eight hours, if the menstruum had been frequently repeated under the regular influence of human temperature.

Exp. 19.—A fragment of the same species with the above, weighing twelve grains, was immersed, for forty-eight hours, in three ounces of distilled water, without addition; temperature from 60 to near 100 degrees. After being taken out, and dried, it was found to have lost one grain three-quarters, became so friable as to crumble, and the solution to precipitate with a few drops of pure ammonia. This species of calculus, therefore, is soluble in water, at temperatures even inferior to that of the human. It is unnecessary I should enter into a further detail of experiments made upon calculi of the mixed kind, having the uric acid, phosphate of ammonia, and sometimes, though rarely, phosphate of lime, intermixed in their strata. Suffice it to say, that the very dilute marine acid speedily takes up the earthy phosphates, leaves the laminæ of the uric acid bare and distinct, ready to crumble, and of easy solution in the weakest alkaline lixivia, and still more so in in lime-water:—a most important consideration in a practical point of view.

It would be trespassing too much on the already tried indulgence of the Reader, to go further into the detail of the circumstances necessary to be attended to, and acquainted with, to ensure success in the application of these principles. These are already tolerably well detailed in the Connoissances Chimiques. To the gentlemen professors in the School of Surgery it more particularly belongs; and from the zeal and talents now in full activity there, what may not be expected? Created only the other day, by a Cleghorn, (a name as deservedly as universally revered;) fostered, afterwards, by the anxious care and talents of Mr. Dease; we find it already arrived at a state of perfect maturity, and holding out to the student advantages no where to be rivalled, if indeed equalled; and that nothing may be wanting to a complete medical as well as surgical education, establishing a chair of botany, supported by the acknowledged abilities of Dr. Wade, both as a botanist and teacher. From the above experiments and observations we may presume to draw the following conclusions:

That acids, and ascescent drinks of all kinds, give rise to gravelly and calculous affections, by causing a separation and precipitation of the native uric acid of urine within the body. That all acids, vegetable or mineral, nay, the native phosphoric acid of urine, in excess, are equally productive of this effect; the tartaric, perhaps, somewhat more so. That, on the other hand, we find lime, both the fixed alkalies, pure as well as aërated, (even in the smallest proportions,) serviceable in these disorders, by uniting with, and keeping in solution, this acid substance. That they also, in the smallest proportions, and diluted state, exert strong solvent powers on this acid in its aggregate form of calculus, provided their action be favoured by degrees of temperature approaching to the human. That, under the same circumstance, contrary to what was generally supposed, the carbonated, sub-carbonated, nay, the super-carbonated, exert similar influence, though in an inferior degree. That lime, even in the small proportion it presents itself to us in lime-water, is a most active and safe solvent of calculi of the uric acid kind, and its various combinations; as has been long since ascertained by Whyte. That, weight for weight, it exceeds even the caustic alkali in any state of dilution that the latter can be applied to the living body. That, finding four ounces of limelime-water, containing only two grains three-quarters, take up, or detach, seven grains three quarters from a very compact calculus, we may be led to suppose this may arise from its action on the agglutinating medium, its affinity to. and energy on, animal matter being so well known; and, if so, may we not expect something from its power on the mulberry calculus, our most formidable enemy? For, though it cannot touch the oxalate of lime, it may the cementing medium, with which it peculiarly abounds.

For the application of these established facts to useful purposes, I must refer to my surgical friends, being all now possessed of the necessary degree of chemical acquirement; and I am happy to find this career already entered on by my friend Mr. Crampton, who has favoured us with an analysis of a pulmonary calculus in the Philosophical Transactions, and from whose professional as well as scientific talents we have every thing to expect in fulfilling (even on this occasion) his duties as a teacher.

Having now endeavoured to accomplish the chief object of this Essay, which was, to establish experimentally a more clear and comprehensive view of the nature of these maladies, and the remedies employed to combat them, than we hitherto possessed, I should not have trespassed further on the time of the Reader, were it not properly suggested, by my friend Dr. Clarke, that it would be of importance to ascertain how far the facts and notions, brought forward in it, may stand confirmed or contradicted by the result of our practical application of them in Simpson's Hospital; an establishment affording the best and most extensive field of observation, of this kind, of any in Europe, that of Luneville, perhaps, excepted.

The benefit of this charity extends equally to the blind and gouty. In the year 1795 I found it to contain thirty-two of the latter; and since that period thirty-four have been admitted: in all, sixty-six gouty patients. Of these the greater number have either complained of gravel, or passed it without any previous or concomitant inconvenience: a circumstance which I had every day occasion to observe, whilst attending to the state of gouty urine. Among the blind and gouty, however, we may count about twenty-two as specifically more afflicted, having occasionally complained of marked and distinct symptoms of this disorder. Of these, we find sixteen among the gouty, and six only among the blind. Now, as the severity of gout is uniformly diminished, nay, in many instances, the disease entirely removed, by a residence of a few years only in the the house, we must expect to find the same take place with respect to gravel, to which it is so strongly and nearly connected. And this singular alleviation of both diseases we can only attribute to the influence of temperance, and the manner of living, very opposite to that of their former habits. The diet in our house consists of bread and milk for breakfast and supper; beef, or mutton, with table beer, for dinner; all of the best quality, and administered with the greatest propriety and regularity; whilst the introduction of ardent spirits is prohibited, and sobriety enforced, by the strict discipline of the house. On the other hand, we find that, previous to their admission, they were either addicted to intemperance, or in the habit, at least, of muddling in public houses, where, after a libation with porter, they indulged in the free use of acidulated punch (the constant nocturnal practice of our middling tradesmen and shop-keepers, who furnish the greatest proportion of our patients). The keeper of a porter-house of considerable resort informs me, that, to please the generality of his customers, he finds it necessary to add the juice of an entire lemon to about two quarts of punch; and that, from this circumstance, he would have experienced a considerable diminution of his profits, if he did not occasionally substitute cream of tartar, or the dilute sulphuric acid: an innocent and safe practice, in his opinion. Now, so satisfied are our patients of the pernicious effects of acids of all kinds, that we find many of them refuse to make use of our table beer during the summer months, through the apprehension of its acescent quality (as was before observed), and which continued to be the practice of Hewson, Khensk, Clapham, and others, for years back: nor do our present two greatest sufferers, Sing and Cox, venture on it at any season but with the greatest caution.

To a removal, then, from the former occasional causes we may attribute no small share of the alleviation of those diseases which takes place with us: a practical observation, that cannot be too generally known. But to return to my subject:—On the slightest appearance of gravelly symptoms, unconnected with fever, or inflammatory tendency of the urinary system, our patients have recourse to an alkaline medicine, the gravelly pills (as they term them), which consist of desiccated soda, in the most convenient form for hospital practice, as well as most suited to gouty stomachs. Of this (as first advised by Beddoes,) one drachm, with the addition of a few grains of capsicum, or drops of essential oil, and the necessary quantity of of hard soap, or extract, is made into twenty pills. Of these, from three to six, or more, are taken in the twenty-four hours; and are found sufficient, not only to alleviate or remove these complaints, but even to render the interference of the physician but seldom necessary. We have had also occasion to remark, that several of our patients, induced by their marked beneficial effects, carried these pills about them, so as to have occasional recourse to them, without much attention to either dose or number.

To this practice, then, we would be disposed to attribute the very pleasing and interesting consideration, that, among so many gravelly patients, there has not occurred, in the course of ten years, a single operation of lithotomy; nor has the catheter, even in the hands of our expert and able surgeon, Mr. Macklin, been able to discover the smallest occasion for it. We could therefore have no opportunity of ascertaining the efficacy of injections into the bladder, as recommended by Whyte, Fourcroy, and myself.

I shall conclude by observing, that it would be interesting to have it in our power to extend these researches to the urine of those who live habitually on different aliment and drinks, particularly of the acescent kind, as well as to that of those who drink waters with mineral alkaline impregnations. But this desirable object can be only obtained by the concurrent exertions and attention of gentlemen of the faculty in different countries and situations. In private practice it is not to be expected; for here, wherever experiment is surmised to be the object, mistrust and suspicion take place of professional confidence. The use of the nitric acid in our venereal hospital, I hoped, would afford some useful facts as to its effects upon the saline contents of urine; the uric acid in particular. But I had not, as yet, sufficient leisure for that inquiry; nor could I, hitherto, obtain the urine of those using it, with all the circumstances necessary to enable me, at this moment, to draw any direct conclusions from my examination of it. In many instances, a morbid state of the urinary system (the urethra in particular) took place. In others, the combined effects of mercury interfered: and in all, no certainty of its not being blended with the urine of others not using this acid. I could not, however, help observing, that the few specimens sent to me, agreed in one particular, viz. their exceeding very little, if at all, the usual healthy standard of acidity. This circumstance must excite our attention the more forcibly, when we consider, sider, that two drachms of nitric acid, nay, sometimes three, diluted in the proportion of one pint of water to each drachm of acid, were taken daily; whilst, on the other hand, a few drops of the acid elixir of vitriol, or tincturæ martis in sp. salis, nay, the weak vegetable acids, and cream of tartar, persevered in for a few days, impart an additional degree of acidity to the urine. Would not this observation (if founded), conjoined with the easy decomposable nature of the acid itself, and its action on animal matter, induce us to lean to the opinion of those who have already asserted that this acid is partly decomposed in the system, imparts its oxygen to it, and that, perhaps, to a degree capable of annulling or destroying its properties as an acid?

And it may be here further remarked, in confirmation of such notion, that those gentlemen most conversant with it here, as well as most capable of judging, entertain strong doubts of its supposed diuretic effects, allowance being made for the necessary quantity of its watery vehicle. If it be, then, truly deoxygenated in the system, why be deterred by its failure, as a radical cure of syphilis, from extending our trials with it here, to other chronic diseases, as they have already done in India?

